# Burden of lower respiratory infections in the Eastern Mediterranean Region between 1990 and 2015: findings from the Global Burden of Disease 2015 study

**DOI:** 10.1007/s00038-017-1007-0

**Published:** 2017-08-03

**Authors:** GBD 2015 Eastern Mediterranean Region Lower Respiratory Infections Collaborators, Maziar Moradi-Lakeh, Charbel El Bcheraoui, Raghid Charara, Ibrahim Khalil, Ashkan Afshin, Nicholas J. Kassebaum, Michael Collison, Farah Daoud, Adrienne Chew, Kristopher J. Krohn, Danny Colombara, Rebecca Ehrenkranz, Kyle J. Foreman, Joseph Frostad, William W. Godwin, Michael Kutz, Puja C. Rao, Robert Reiner, Christopher Troeger, Haidong Wang, Amanuel Alemu Abajobir, Kaja M Abbas, Semaw Ferede Abera, Laith J. Abu-Raddad, Kelemework Adane, Aliasghar Ahmad Kiadaliri, Alireza Ahmadi, Muktar Beshir Ahmed, Ayman Al-Eyadhy, Khurshid Alam, Noore Alam, Deena Alasfoor, Reza Alizadeh-Navaei, Fatma Al-Maskari, Rajaa Al-Raddadi, Ubai Alsharif, Khalid A. Altirkawi, Nahla Anber, Hossein Ansari, Carl Abelardo T. Antonio, Palwasha Anwari, Hamid Asayesh, Solomon Weldegebreal Asgedom, Tesfay Mehari Atey, Euripide Frinel G. Arthur Avokpaho, Umar Bacha, Aleksandra Barac, Shahrzad Bazargan-Hejazi, Neeraj Bedi, Zulfiqar A. Bhutta, Michael Brauer, Zahid A Butt, Carlos A. Castañeda-Orjuela, Hadi Danawi, Shirin Djalalinia, Aman Yesuf Endries, Babak Eshrati, Maryam S. Farvid, Seyed-Mohammad Fereshtehnejad, Florian Fischer, Alberto L. Garcia-Basteiro, Kiros Tedla Gebrehiwot, Tsegaye Tewelde Gebrehiwot, Gessessew Bugssa Hailu, Randah Ribhi Hamadeh, Mitiku Teshome Hambisa, Samer Hamidi, Mohammad Sadegh Hassanvand, Mohammad T. Hedayati, Nobuyuki Horita, Abdullatif Husseini, Spencer Lewis James, Mehdi Javanbakht, Jost B Jonas, Amir Kasaeian, Yousef Saleh Khader, Ejaz Ahmad Khan, Gulfaraz Khan, Abdullah Tawfih Abdullah Khoja, Jagdish Khubchandani, Yun Jin Kim, Niranjan Kissoon, Heidi J. Larson, Asma Abdul Latif, Cheru Tesema Leshargie, Raimundas Lunevicius, Hassan Magdy Abd El Razek, Mohammed Magdy Abd El Razek, Reza Majdzadeh, Azeem Majeed, Reza Malekzadeh, Habibolah Masoudi Farid, Alem Mehari, Ziad A. Memish, Desalegn Tadese Mengistu, Haftay Berhane Mezgebe, Sachiko Nakamura, Eyal Oren, P. A. Mahesh, Farshad Pourmalek, Mostafa Qorbani, Amir Radfar, Anwar Rafay, Vafa Rahimi-Movaghar, Rajesh Kumar Rai, David Laith Rawaf, Salman Rawaf, Amany H. Refaat, Satar Rezaei, Mohammad Sadegh Rezai, Hirbo Shore Roba, Gholamreza Roshandel, Mahdi Safdarian, Saeid Safiri, Mohammad Ali Sahraian, Payman Salamati, Abdallah M. Samy, Benn Sartorius, Sadaf G. Sepanlou, Masood Ali Shaikh, Morteza Shamsizadeh, Mika Shigematsu, Jasvinder A. Singh, Muawiyyah Babale Sufiyan, Arash Tehrani-Banihashemi, Mohamad-Hani Temsah, Roman Topor-Madry, Olalekan A. Uthman, Stein Emil Vollset, Fiseha Wadilo, Tolassa Wakayo, Andrea Werdecker, Tissa Wijeratne, Mohsen Yaghoubi, Hassen Hamid Yimam, Naohiro Yonemoto, Mustafa Z. Younis, Maysaa El Sayed Zaki, Aisha O. Jumaan, Theo Vos, Simon I. Hay, Mohsen Naghavi, Christopher J. L. Murray, Ali H. Mokdad

**Affiliations:** Institute for Health Metrics and Evaluation, 2301 5th Avenue, Suite 600, Seattle, WA 98121 USA

**Keywords:** Lower respiratory infection, Incidence, Mortality, DALY, Eastern Mediterranean Region

## Abstract

**Objectives:**

We used data from the Global Burden of Disease 2015 study (GBD) to calculate the burden of lower respiratory infections (LRIs) in the 22 countries of the Eastern Mediterranean Region (EMR) from 1990 to 2015.

**Methods:**

We conducted a systematic analysis of mortality and morbidity data for LRI and its specific etiologic factors, including pneumococcus, *Haemophilus influenzae* type b, Respiratory syncytial virus, and influenza virus. We used modeling methods to estimate incidence, deaths, and disability-adjusted life-years (DALYs). We calculated burden attributable to known risk factors for LRI.

**Results:**

In 2015, LRIs were the fourth-leading cause of DALYs, causing 11,098,243 (95% UI 9,857,095–12,396,566) DALYs and 191,114 (95% UI 170,934–210,705) deaths. The LRI DALY rates were higher than global estimates in 2015. The highest and lowest age-standardized rates of DALYs were observed in Somalia and Lebanon, respectively. Undernutrition in childhood and ambient particulate matter air pollution in the elderly were the main risk factors.

**Conclusions:**

Our findings call for public health strategies to reduce the level of risk factors in each age group, especially vulnerable child and elderly populations.

**Electronic supplementary material:**

The online version of this article (doi:10.1007/s00038-017-1007-0) contains supplementary material, which is available to authorized users.

## Introduction

Lower respiratory infections (LRIs) are one of the leading causes of death and disability-adjusted life-years (DALYs) worldwide, especially among children under 5 (Kassebaum et al. 2016; Wang et al. 2016). Since 1990, LRI has been the closest competitor of cardiovascular diseases as the top leading cause of DALYs in the Eastern Mediterranean Region (EMR) and the Arab world (Mokdad et al. 2014, 2016). Several factors, such as poverty, indoor and outdoor air pollution, malnutrition, smoking, chronic lung diseases, and delayed and inappropriate case management contribute to the high burden of LRI (Hadi 2003). Population-based measures of morbidity and mortality of LRI are scarce. Most of the available data are limited to children and are based on modeling approaches. Even fewer data are available for causative agents of LRI (Kovacs et al. 2015).

Rudan et al. estimated an incidence of 0.22 episodes of community-acquired pneumonia per child-year in children under 5 in low- and middle-income countries in 2010 (Rudan et al. 2013) The Child Health Epidemiology Reference Group (CHERG) estimated 0.26 episodes per child-year for the world and 0.28 for the World Health Organization’s (WHO) Eastern Mediterranean Region (EMR). These estimates translate to about 20 million cases of childhood pneumonia each year in the EMR, with approximately 10% of cases requiring hospitalization (Rudan et al. 2008). More than 99% of all LRI deaths occur in low- and middle-income countries. Although about 62% of children with severe LRI reach hospitals, more than 80% of all childhood LRI deaths take place outside the hospital setting (Nair et al. 2013; Tong 2013). Despite high levels of morbidity and mortality, the decreasing trend in LRI mortality rates has contributed to increasing life expectancy worldwide (Wang et al. 2016). Improvements in nutritional status (less malnutrition through attention to childhood and maternal nutritional status), increased uptake of vaccines such as immunization against *Streptococcus pneumoniae* (pneumococcus) and *Haemophilus influenzae* type b (Hib) in children and high-risk populations in EMRs, and improved access to antibiotics and supportive care have decreased the incidence and fatality of LRI in many countries (Williams and Shah 2012; Tong 2013).

In this study, we report findings from the Global Burden of Diseases, Injuries, and Risk Factors Study 2015 (GBD 2015) on LRI in the 22 countries of the EMR between 1990 and 2015. We describe the burden of LRI and a subset of specific etiologic pathogens, including pneumococcus, Hib, respiratory syncytial virus (RSV), and influenza virus, based on deaths, years of life lost (YLLs), years lived with disability (YLDs), and disability-adjusted life-years (DALYs).

## Methods

GBD 2015 covers 195 countries, 21 regions, and seven super-regions from 1990 to 2015 for 315 diseases and injuries and 79 risk factors. A counterfactual approach was used for estimating LRI etiologies, one of the methodological differences compared to GBD 2010. Detailed descriptions of the methodological approach for GBD 2015 have been published elsewhere (Forouzanfar et al. 2016; Kassebaum et al. 2016; Vos et al. 2016; Wang et al. 2016).

The EMR consists of 22 countries with different levels of gross national income (GNI) per capita. The low-income countries (LICs) are Afghanistan, Djibouti, Somalia, and Yemen. The middle-income countries (MICs) are Egypt, Iran, Iraq, Jordan, Lebanon, Libya, Morocco, Pakistan, Palestine, Sudan, Syria, and Tunisia. The high-income countries (HICs) are Bahrain, Kuwait, Oman, Qatar, Saudi Arabia, and the United Arab Emirates (UAE).

All types of LRI (bronchitis, bronchiolitis, and pneumonia) were included in this study. We used the following International Classification of Diseases (ICD-10) codes (or their corresponding codes from earlier ICD versions) as equivalent to LRI: J09–J15.8, J16–J16.9, J20–J21.9, P23-P23.9, and Z25.1. The ICD-10 codes for etiologic categories of LRI included J09–J11.89 and Z25.1 for Influenza virus, J12.1 for RSV, J13, J13.0, J15.3, J15.4, and J15.6 for pneumococcus, J14 and J14.0 for Hib, and J12, J12.0, J12.2–J12.9, J15–J15.9, J15.5, J15.7, J15.8, J16–J16.9, J20–J21.9, and P23-P23.9 for other LRI. We did not include tuberculosis in this study; it has been classified as a separate item in GBD.

An analysis of available data on all-cause mortality for all countries was undertaken (Wang et al. 2016). For pathogen-specific mortality rates of LRI, we used a counterfactual approach based on the epidemiological concept of attributable mortality. The change in LRI was estimated assuming the condition that a specific pathogen was not present. We adopted different approaches to estimate bacterial and viral causes based on the available data. For pneumococcal and Hib LRI, we estimated the causal fraction from vaccine efficacy trials data. For RSV and influenza, we relied on observational studies that measured causal fractions among hospital admissions for LRI (Vos et al. 2016; Wang et al. 2016). We estimated the causal fractions among cases by country, age, and sex. To account for the higher case-fatality rate of bacterial versus viral LRI, we applied a relative case-fatality differential based on hospital data that included cases coded to the specific pneumonia causes. Our mortality estimates were used to calculate cause-specific YLLs for each age, sex, location, and calendar year.

To estimate LRI-related morbidity, a systematic review of studies on epidemiological indicators of LRI was done as part of the GBD standard methodology. We used 197 sources of data from EMR countries (46 for non-fatal outcomes and others for cause of death) which contained data on LRI. A list of all data sources is available on the Institute for Health Metrics and Evaluation’s website. (Institute for Health Metrics and Evaluation) A series of Bayesian meta-regression analyses through DisMod-MR 2.1 were used for disease modeling. Model-based epidemiological estimates in combination with disability weights were used to calculate cause-specific YLDs for each age, sex, location, and calendar year. DALYs were calculated through summation of YLLs and YLDs (Kassebaum et al. 2016; Vos et al. 2016).

We estimated burden of LRI attributable to childhood malnutrition (underweight, wasting, and stunting), non-exclusive breastfeeding, zinc deficiency, smoking, secondhand smoke exposure, household air pollution from solid fuels, and ambient air pollution as known LRI risk factors. Details on definitions and measures of age- and sex-specific relative risks of LRI for each of the risk factors are available elsewhere (Forouzanfar et al. 2016).

We report 95% uncertainty intervals (UI) for each estimate—such as rates or numbers of deaths or DALYs. We estimated UIs by taking 1000 samples from the posterior distribution of each quantity and using the 25th- and 975th-ordered draws of the uncertainty distribution.

## Results

LRI deaths decreased by 30.2% from 273,714 (95% UI 239,890–306,648) in 1990 to 191,114 (95% UI 170,934–210,705) in 2015 in EMR countries. In spite of the decrease, LRI is still the third-leading cause of death for all ages in 2015. The death rate per 100,000 was 74.0 (95% UI 64.8–82.9) in 1990 and decreased to 29.5 (95% UI 26.4–32.5) in 2015, a 60.1% reduction. The age-standardized death rate for LRI was 65.4 (95% UI 58.6–73.4) in 1990 and declined by 38.5% to 40.2 (95% UI 36.0–45.2) in 2015. In 2015, 4.8% (95% UI 4.3–5.2) of all deaths and 13.0% (95% UI 11.5–14.7) of under-5 deaths were due to LRI.

At the regional level, the highest death rates and numbers of deaths were among children under 5 years old, followed by those aged 65 years or older. However, there was considerable heterogeneity in the age pattern of LRI deaths between the countries of the EMR. In all high-income countries (Bahrain, Saudi Arabia, Kuwait, Oman, Qatar, and UAE), and some of the middle-income countries (Iran, Lebanon, Libya, Morocco, Palestine, Syria, and Tunisia), the LRI death rates among older age groups were greater than the mortality rates among children under 5 years. Death rates were 31.5 per 100,000 (95% UI 27.3–35.4) in men compared to 27.4 per 100,000 in women (95% UI 23.9–31.2) in the region. The LRI death rate was not different in EMR countries in boys versus girls under 5 (2015).

**Fig. 1:**
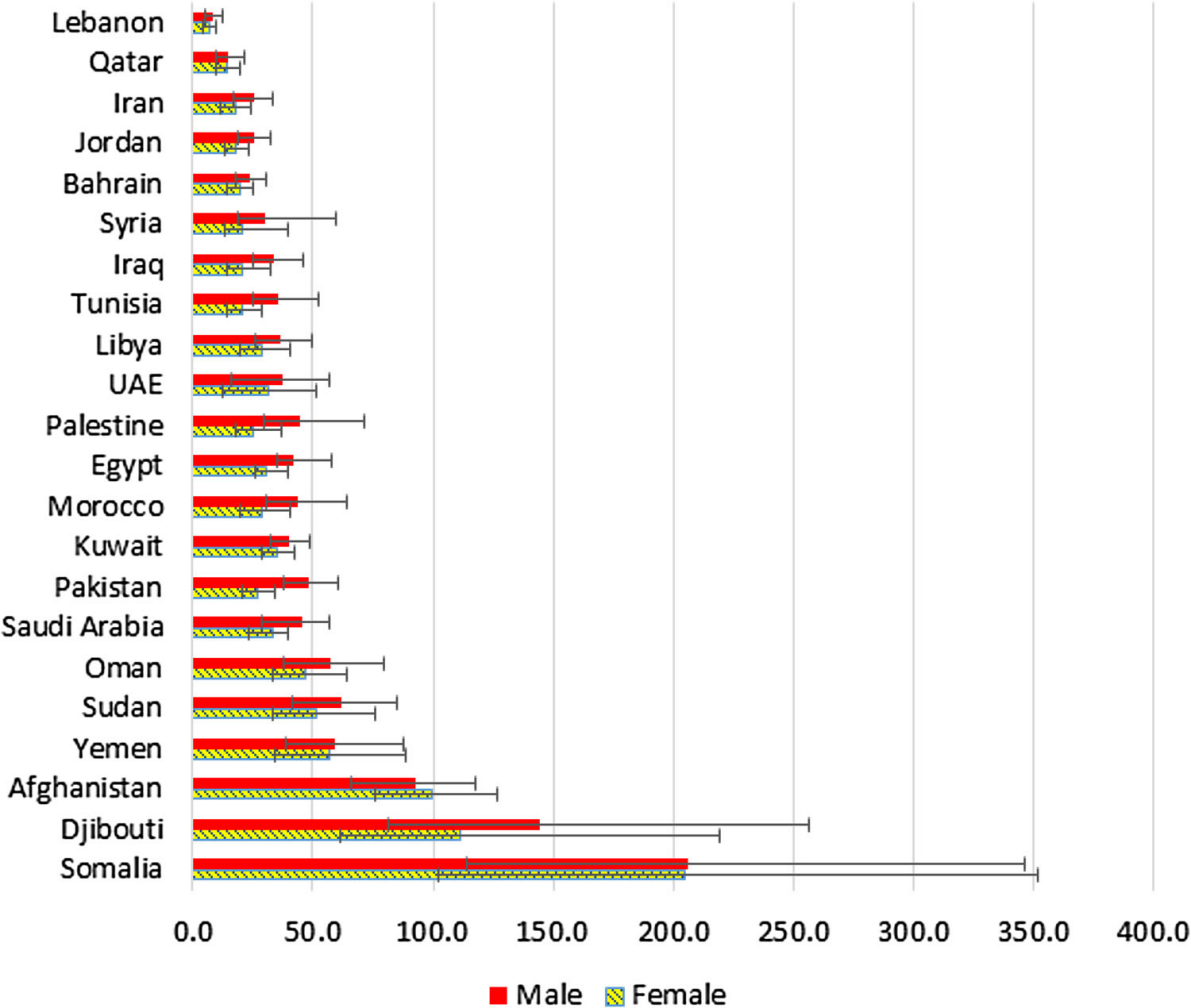
Age-standardized death rates of lower respiratory tract infections per 100,000 population in the countries of the Eastern Mediterranean Region, Global Burden of Disease study, 2015

Figure 1 shows the age-standardized death rates for LRI by EMR country in 2013. Somalia, Djibouti, and Afghanistan had the highest death rates in the region.

**Fig. 2:**
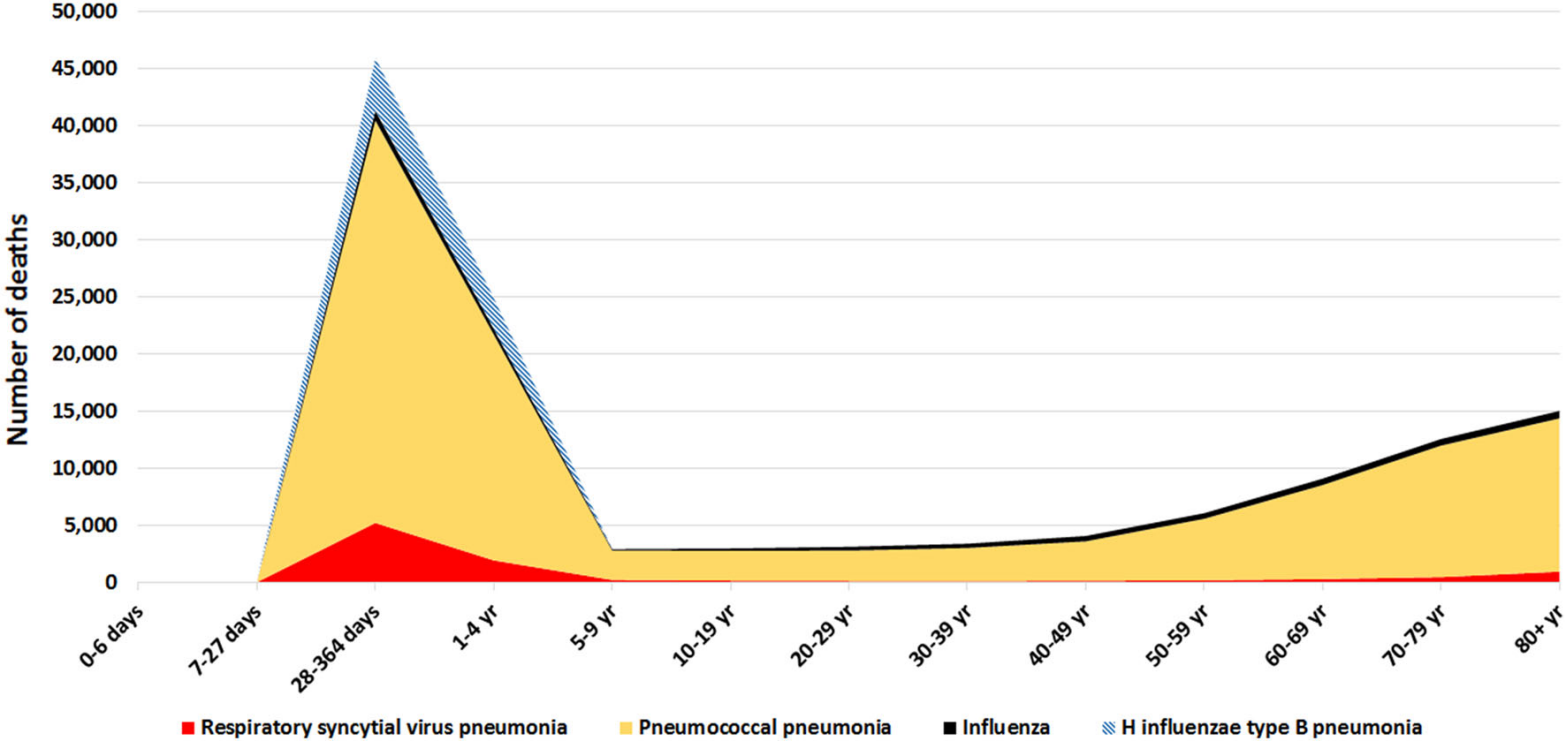
Number of lower respiratory tract infection deaths by etiologic causes in the Eastern Mediterranean Region, Global Burden of Disease study, 2015

Among the etiologic causes, pneumococcus had the highest mortality rate (16.6 per 100,000, 95% UI 10.0–22.9), followed by respiratory syncytial virus (1.5 per 100,000, 95% UI 0.9–2.4), *Haemophilus influenzae* type b (1.1 per 100,000, 95% UI 0.0–2.3), and influenza virus (0.8 per 100,000, 95% UI 0.5–1.2). Figure 2 demonstrates the contribution of different etiologic causes to LRI deaths by age.

The rate of YLLs per 100,000 population decreased by 69.5% from 5579 (95% UI 4,831–6,320) in 1990–1702 (95% UI 1,510–1,902) in 2015. However, LRI was still the third-leading cause of YLLs in 2015.

e-Table 1 shows age-standardized incidence rates of LRI in males and females of the EMR countries. The highest incidence rate was observed in Afghanistan and the lowest in Tunisia for both sexes. In all countries but Afghanistan, Bahrain, and Djibouti, the incidence rates were higher among men than women, with the highest male-to-female ratio in Iran and Libya.

The rate of YLDs per 100,000 decreased from 15.8 (95% UI 10.7–22.9) to 9.7 (95% UI 6.5–13.6) per 100,000 during 1990–2015.

Total DALYs from LRI decreased by 40.6%, from 20,746,747 (95% UI 17,954,899–23,444,142) in 1990 to 11,098,243 (95% UI 9,857,095–12,396,566) in 2015. LRI, which was the leading cause of DALYs in 1990, was ranked fourth in 2015, behind ischemic heart disease, neonatal preterm birth complications, and neonatal encephalopathy. LRI DALY rates were 5594 (95% UI 4851–6334) and 1712 (95% UI 1520–1912) DALYs per 100,000 population in 1990 and 2015, respectively, a 69.4% reduction. About 4.9% (95% UI 4.3–5.5) of DALYs in all ages and 12.2% (95% UI 10.8–13.8) of DALYs in children under 5 were due to LRI in 2015.

**Fig. 3:**
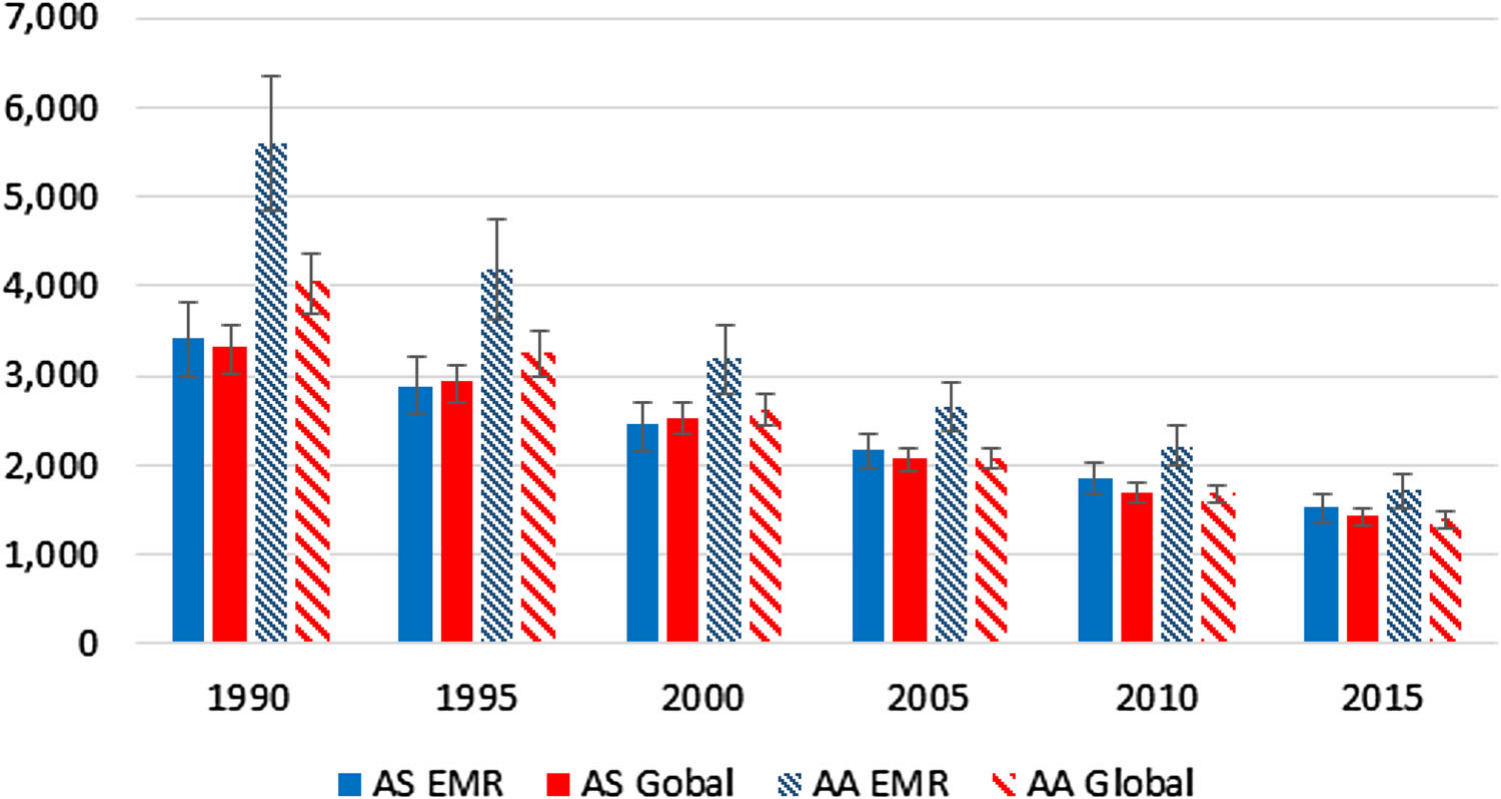
Trends of age-standardized (AS) and all-age (AA) DALY rates of lower respiratory infections in the Eastern Mediterranean Region and the world, Global Burden of Disease study, 1990–2015

Age-standardized DALY rates were 3411 per 100,000 (95% UI 2,993–3,824) in 1990 compared to 1518 per 100,000 (95% UI 1357–1673) in 2015 in the EMR. Global age-standardized DALY rates for LRI were lower than those in the EMR: 3310 (95% UI 3033–3551) in 1990, and 1428 (95% UI 1330–1511) in 2013. There was a 56.9% reduction in age-standardized DALY rates at the global level, which was similar to the EMR (55.5% reduction) during 1990–2015. Figure 3 presents trends in age-standardized and all-ages LRI DALY rates between 1990 and 2015 globally and for the EMR.

**Fig. 4:**
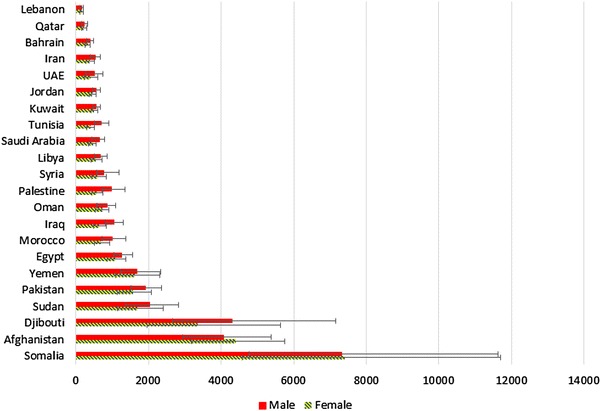
Age-standardized disability-adjusted life-years (DALYs) rates of lower respiratory tract infections per 100,000 population in the countries of Eastern Mediterranean Region, Global Burden of Disease study, 2015

Figure 4 shows age-standardized LRI DALY rates in the EMR countries. The highest rates were seen in Somalia, Afghanistan, and Djibouti. LRI DALY rates did not differ significantly between males and females.

**Fig. 5:**
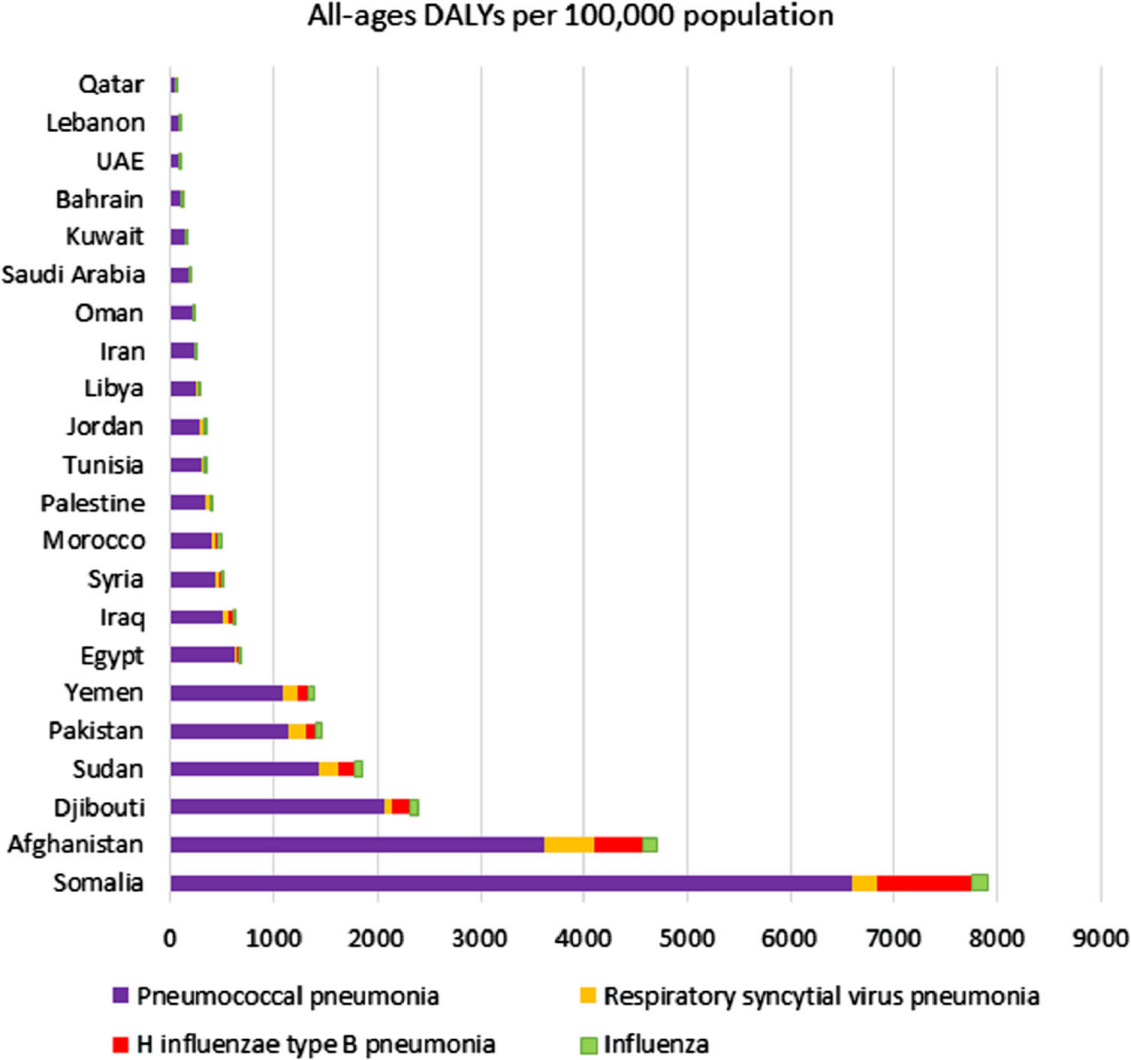
Disability-adjusted life-years (DALYs) rates for different etiologies of lower respiratory tract infections in the countries of the Eastern Mediterranean Region, Global Burden of Disease study, 2015

Figure 5 shows DALY rates for different etiologies of LRI in EMR countries in 2015. The largest variation in country-specific DALYs (highest to lowest ratio) was observed for Hib (2068.7) compared to 125.5 for pneumococcus, 109.4 for RSV, and 30.6 for influenza. The DALY rate for Hib was less than one DALY per 100,000 in UAE and Qatar, compared to 916.7 in Somalia and 458.5 in Afghanistan (Fig. 5).

**Fig. 6:**
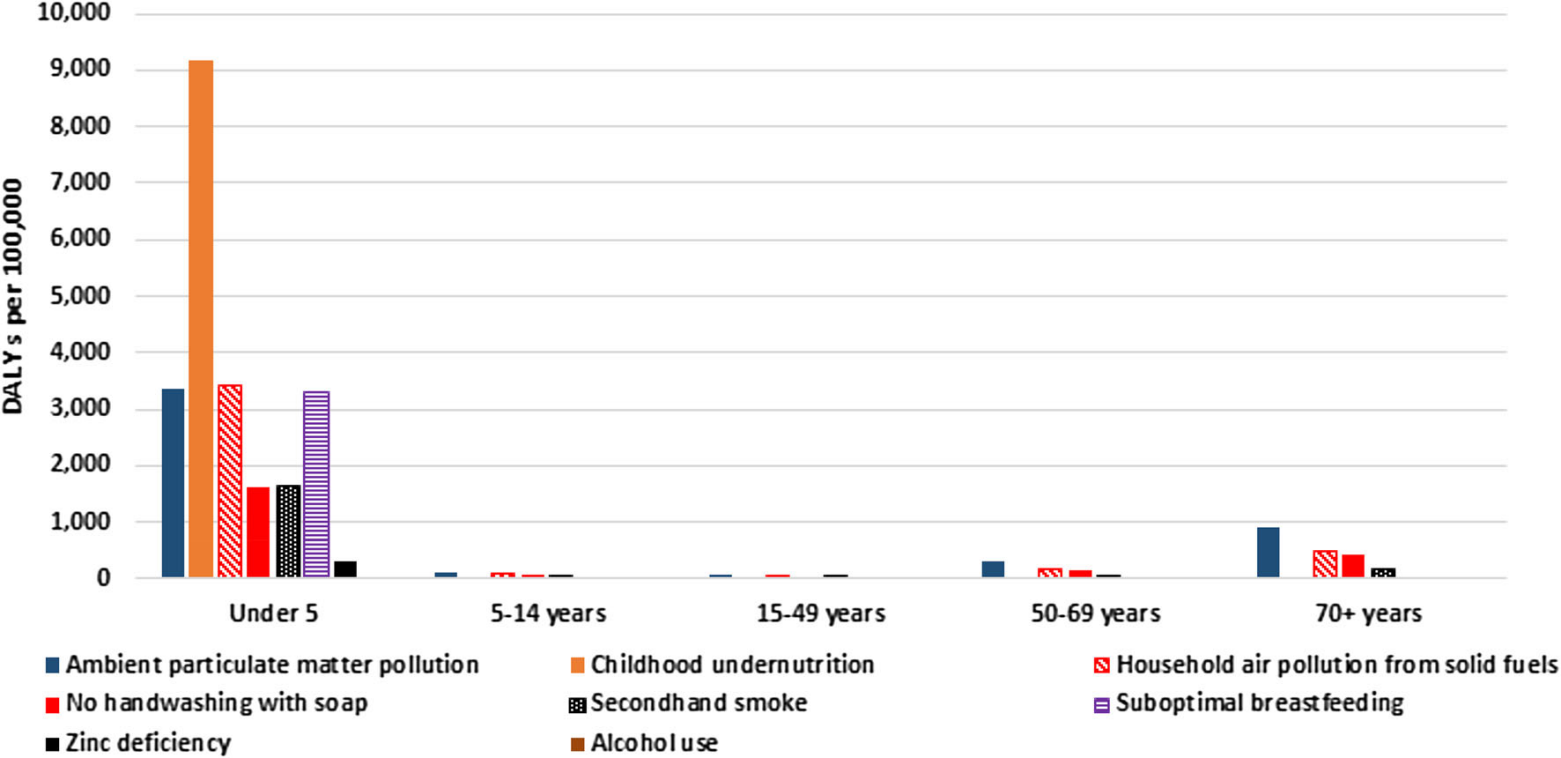
Disability-adjusted life-years (DALYs) attributable to risk factors of lower respiratory tract infections, Eastern Mediterranean Region, Global Burden of Disease study, 2015

Figure 6 shows the LRI DALY rates attributable to different risk factors in 2015. Childhood undernutrition, household air pollution from solid fuels, ambient particulate matter pollution, suboptimal breastfeeding, secondhand smoke, no handwashing with soap, and zinc deficiency were the most important risk factors for LRI in children under 5. Among those aged 70 years or older, ambient particulate matter pollution, household air pollution from solid fuels, smoking, no handwashing with soap, secondhand smoke, and alcohol use were the main risk factors.

## Discussion

Our study showed a substantial burden of LRI in the EMR. Despite a decline in the burden of LRI since 1990, LRI remained the fourth-leading cause of DALYs and third-leading cause of death in the region in 2015. The all-ages burden of LRI in the EMR is considerably higher than global rates, and even the age-standardized burden is slightly higher. Our findings call for intensified efforts to control and reduce the burden of LRI in the EMR, especially among low-income countries and low-resource settings. Moreover, since the current situation of health status and LRI varies widely between the EMR countries, suggested interventions to avert LRI deaths and disabilities should be specified at the national or even subnational level (Akseer et al. 2015).

The unstable political situation and wars in many of the EMR countries present a major challenge for controlling communicable diseases in the region (Haq et al. 2013). Countries with highest burden of LRI (Somalia, Afghanistan, and Djibouti) have been involved in armed conflicts and social unrest in recent decades. Conflict-affected children experience significant increases in deaths due to diarrheal diseases, malnutrition, respiratory infections, and measles (Guha-Sapir and Panhuis 2004). These are due to different causes such as displacement of populations, breakdown of health and social services, and increased risk of disease transmission (Murray et al. 2008). Beyond improving social determinants that affect LRI, a combination of public health and clinical strategies is required to control them (World Health Organization and UNICEF 1986). The “Protect, Prevent, and Treat” framework provides a blueprint for such interventions (Qazi et al. 2015). The need for an integrated global action plan led to the launch of the Global Action Plan for the Prevention and Control of Pneumonia and Diarrhea (GAPPD), which can be used by EMR countries as well (Qazi et al. 2015).

Immunization is one of the most effective public health strategies to prevent and reduce the burden of LRI, such as immunization against pneumococcal infection, Hib, and seasonal influenza. Immunization is highly effective at reducing the burden of ill health from pneumococcal infection and Hib, but less so for influenza. The effectiveness of influenza vaccination depends on the match between the antigenic strains included in the vaccine and the circulating strains in the community, and this can vary substantially from year to year. EMR countries are at different stages of adopting these vaccines. High-income EMR countries have introduced pneumococcal conjugate vaccine (PCV) to their national immunization programs. Moreover, all low-income countries in the region except Somalia have introduced PCV thanks to support from Gavi, the Vaccine Alliance. (Gavi 2017 the Vaccine Alliance) Somalia has issues with under-coverage of immunization, even for traditional vaccines such as DTP vaccine (diphtheria, tetanus, and pertussis). The majority of middle-income countries in the region (Egypt, Iran, Iraq, Jordan, Lebanon, Syria, and Tunisia), have yet to introduce PCV or are in the early stages of planning (Moradi-Lakeh and Esteghamati 2013; Sibak et al. 2015).

All EMR countries have introduced Hib vaccine to their immunization programs, and coverage of Hib3 in the EMR has increased from 13% in 2005 to 72% in 2014. However, coverage of Hib3 varies across EMR countries (World Health Organization 2017). Hib3 coverage was 75% or less in five countries in 2014: Somalia, Syria, Iraq, Pakistan, and Afghanistan (World Health Organization 2017). The coverage rate in Syria dropped from 80% in 2010 to 43% due to the ongoing war in the country (World Health Organization 2017). Iran was the last country in the region to introduce Hib vaccine in late 2014 and has not yet published coverage data. Economic sanctions against Iran had an impact on health policies and slowed down implementation of immunization programs and policies (Kheirandish et al. 2015; Massoumi and Koduri 2015).

There are different immunization policies against seasonal influenza virus in EMR countries. Although the impact of the influenza vaccine on prevention of LRI is not as clear as that of conjugate vaccines against pneumococcal infections and Hib, its effectiveness in protecting specific high-risk groups against severe influenza-associated disease and death is generally accepted (Voordouw et al. 2003; Nichol et al. 2007). Indeed, the policies for influenza vaccination are impacted by national capacities, resources, and epidemiological status (World Health Organization 2012). In the countries that have already established vaccination programs, a number of strategies such as public education and reminders for parents and immunization providers can increase immunization coverage (Williams et al. 2011).

Improving diet and nutritional status in the population groups that are most vulnerable to LRI, such as children and elderly people, can help prevent infection. Additionally, enhancing breastfeeding and encouraging zinc supplementation can reduce the burden of childhood LRI in the region (Roth et al. 2008, 2010; Shah et al. 2013). Moreover, based on our data, reducing indoor air pollution (mainly due to household use of solid fuels) and outdoor air pollution (especially ambient particulate matter) contribute to reducing the burden of disease in all age groups (Renzetti et al. 2009; Rehfuess et al. 2009). Unfortunately, many countries of the region suffer from air pollution: while Afghanistan, Pakistan, Somalia, Sudan, and Yemen have considerable issues with household air pollution, more countries are affected by outdoor air pollution (Afghanistan, Egypt, Iran, Iraq, Lebanon, Libya, Pakistan, Saudi Arabia, Syria, and Tunisia) (Cohen et al. 2017; World Health Organization Eastern Mediterranean Region 2017). Smoking and exposure to secondhand smoke are other factors that influence LRI (Oberg et al. 2011). Most of the countries of the region (all except Somalia and Morocco) have ratified the Framework Convention on Tobacco Control (FCTC), which provides a baseline for tobacco control (Framework Convention Alliance Parties to the WHO 2017). However, the anti-smoking activities need to be boosted (Usmanova and Mokdad 2013) and expanded to shisha use, which is very common in the region (Moradi-Lakeh et al. 2015).

Access to adequate health care and appropriate case management (rapid diagnosis, access to antibiotics and supportive care) are important therapeutic interventions for LRI control (Bhutta et al. 2013). Although in general health care access has improved in EMR countries, there is still limited access to high-quality health care due to availability, accessibility, acceptability, and costs, especially for those who live in remote areas as well as disadvantaged groups (Kronfol 2012; Takian et al. 2013). The use of simple, standardized guidelines for diagnosis and treatment of LRI reduces complications and deaths. These guidelines should be made available to all care facilities including community health facilities and mobile units (Kronfol 2012; Takian et al. 2013). Unfortunately, many EMR countries have not implemented such evidence-based guidelines. Although the required expertise and local data for developing guidelines are not available in the majority of EMR countries, adaptation of international guidelines can be used as an alternative method to increase quality of care (Rashidian 2008). On the other hand, several countries in the region (such as Libya, Syria, and Yemen, as the most recent examples) have experienced considerable setbacks in access to health care because of war, social unrest, and mass displacement of populations (Dewachi et al. 2014; Burki 2015). Shortages in the provision of acute care, inpatient and outpatient clinical services as well as primary care services, and access to essential medicines are among the most urgent issues in such unstable conditions. Moreover, continuity of providing preventive and health promotion services is difficult during war and instability; several reports have demonstrated the effect of war on childhood malnutrition and the inability to maintain a cold chain for vaccines during the wars in Afghanistan, Iraq, and Yemen (Assefa et al. 2001; Levy and Sidel 2016; Burki 2016). These are among the well-known LRI risk factors.

Surveillance systems are crucial for planning and managing LRI and identifying emerging infections (Breiman et al. 2013). In 2015, several outbreaks of LRI occurred in the EMR, for instance influenza A(H1N1) pdm09 in Libya, Kuwait, and Jordan; avian influenza A (H5N1) in Egypt; and Middle East respiratory syndrome coronavirus (MERS-CoV) in Saudi Arabia and Jordan (WHO Regional Office for the Eastern Mediterranean 2016). However, many EMR countries do not have adequate health information systems to monitor the situation of LRI. (World Health Organization and UNICEF 2013) Therefore, it is important to assist countries to set up such systems to identify and manage outbreaks, and monitor the trends and risk factors of LRI. It also helps to improve accuracy of estimates for EMR in the next rounds of the GBD study.

Our study has some limitations. First, there are not enough original data for all countries of the region. For instance, many countries did not have estimates on incidence or etiologic agents of LRI. However, we used modeling approaches to estimate the burden, even for countries with limited data. While we did not assess avian influenza, severe acute respiratory syndrome (SARS), and MERS-CoV (Beauté et al. 2014; Milne-Price et al. 2014) as separate etiologic agents, all of them were included in total numbers of incidence, deaths, and DALYs.

### Conclusion

LRI is one of the leading causes of morbidity and mortality in the EMR. Efforts are urgently needed to prevent and control LRI in the region, especially in the low-income countries of the region. All countries should consider adopting vaccines against pneumococcus, if they have not already, to reduce the burden. To adequately address the LRI burden, a public health and health care systems approach is needed. A comprehensive plan that includes addressing known risk factors such as poor diet, smoking, and exposure to secondhand smoke; health systems to improve prevention and treatment of cases; and community programs to increase awareness and immunization uptake are needed. A reliable surveillance system is required to monitor trends of disease burden and evaluate new interventions.

Unstable economies, political instability, and unrest in the region are major barriers to improving health. Therefore, it is important for the international community to work to improve political stability in the EMR, and to strengthen support for low-income countries and disadvantaged groups, in order to reduce LRI morbidity and mortality in the region.

## Electronic supplementary material

Below is the link to the electronic supplementary material.

Supplementary material 1 (DOCX 19 kb)

Supplementary material 2 (XLSX 25 kb)
