# Supplementary material for: Burden of lower respiratory infections in the Eastern Mediterranean Region between 1990 and 2015: findings from the Global Burden of Disease 2015 study
Source: Int J Public Health. 2017 Aug 3;63(Suppl 1):97–108. doi: 10.1007/s00038-017-1007-0 (PMC5973986; doi:10.1007/s00038-017-1007-0)
Supplement: Supplementary file 1 — Supplementary material 1 (DOCX 19 kb) [file 38_2017_1007_MOESM1_ESM.docx]

Electronic Supplementary Material

**Article title:**

Burden of lower respiratory infections in the Eastern Mediterranean Region between 1990 and 2015: Findings from the Global Burden of Disease 2015 study

**Journal:**

International Journal of Public Health

**Authors:**

GBD 2015 Eastern Mediterranean Region LRI Collaborators

**Corresponding author:**

Ali H. Mokdad

Institute for Health Metrics and Evaluation, University of Washington, Seattle, WA, United States

Email: [mokdaa@uw.edu](mailto:mokdaa@uw.edu)

e-Table 1. Age-standardized sex-specific incidence rates of lower respiratory infections (percent)

in the countries of the Eastern Mediterranean Region, 2015. (Global Burden of Disease Study 2015, Eastern Mediterranean Countries, 2015)

| Countries | Male | | | Female | | | Both | | |
| --- | --- | --- | --- | --- | --- | --- | --- | --- | --- |
|  | Incidence | 95% UI | | Incidence | 95% UI | | Incidence | 95% UI | |
| Afghanistan | 4.0 | 4.3 | 3.8 | 6.0 | 6.5 | 5.6 | 5.0 | 5.4 | 4.7 |
| Bahrain | 3.8 | 4.0 | 3.5 | 4.4 | 4.7 | 4.2 | 4.1 | 4.3 | 3.8 |
| Djibouti | 7.1 | 7.6 | 6.6 | 8.4 | 9.0 | 7.8 | 7.8 | 8.3 | 7.3 |
| Egypt | 4.2 | 4.5 | 4.0 | 4.7 | 5.0 | 4.4 | 4.5 | 4.7 | 4.2 |
| Iran | 4.1 | 4.4 | 3.9 | 4.8 | 5.1 | 4.5 | 4.4 | 4.7 | 4.2 |
| Iraq | 4.8 | 5.1 | 4.5 | 5.5 | 5.9 | 5.1 | 5.2 | 5.5 | 4.8 |
| Jordan | 4.1 | 4.4 | 3.9 | 4.6 | 4.8 | 4.3 | 4.3 | 4.6 | 4.1 |
| Kuwait | 4.0 | 4.2 | 3.8 | 5.0 | 5.3 | 4.7 | 4.4 | 4.7 | 4.2 |
| Lebanon | 4.2 | 4.5 | 3.9 | 5.0 | 5.4 | 4.7 | 4.6 | 4.9 | 4.3 |
| Libya | 4.3 | 4.6 | 4.0 | 5.5 | 5.8 | 5.1 | 4.9 | 5.2 | 4.6 |
| Morocco | 5.1 | 5.4 | 4.8 | 5.5 | 5.9 | 5.2 | 5.3 | 5.6 | 5.0 |
| Oman | 4.0 | 4.3 | 3.8 | 5.1 | 5.5 | 4.8 | 4.5 | 4.7 | 4.2 |
| Pakistan | 5.6 | 6.0 | 5.3 | 5.2 | 5.6 | 4.9 | 5.4 | 5.7 | 5.1 |
| Palestine | 4.9 | 5.2 | 4.6 | 5.4 | 5.8 | 5.0 | 5.1 | 5.4 | 4.8 |
| Qatar | 3.6 | 3.8 | 3.3 | 4.4 | 4.7 | 4.1 | 3.9 | 4.1 | 3.6 |
| Saudi Arabia | 3.6 | 3.8 | 3.5 | 4.4 | 4.6 | 4.2 | 4.0 | 4.2 | 3.8 |
| Somalia | 7.4 | 7.9 | 6.9 | 8.3 | 8.8 | 7.8 | 7.9 | 8.3 | 7.4 |
| Sudan | 5.9 | 6.4 | 5.5 | 6.9 | 7.5 | 6.4 | 6.4 | 6.9 | 6.0 |
| Syria | 4.6 | 5.0 | 4.3 | 5.4 | 5.8 | 5.0 | 5.0 | 5.4 | 4.7 |
| Tunisia | 3.1 | 3.3 | 2.9 | 3.7 | 3.9 | 3.4 | 3.4 | 3.6 | 3.2 |
| United Arab Emirates | 2.8 | 2.9 | 2.6 | 3.4 | 3.6 | 3.2 | 3.0 | 3.2 | 2.8 |
| Yemen | 7.7 | 8.2 | 7.2 | 8.7 | 9.4 | 8.2 | 8.2 | 8.8 | 7.7 |
